# Blood lead and other metal biomarkers as risk factors for cardiovascular disease mortality: Erratum

**DOI:** 10.1097/MD.0000000000007997

**Published:** 2017-09-01

In the article, “Blood Lead and Other Metal Biomarkers as Risk Factors for Cardiovascular Disease Mortality”,^[1]^ which appeared in Volume 95, Issue 1 of *Medicine*, the sentence “Each corrected variable was multiplied by the weighted geometric mean of hematocrit or hemoglobin, respectively, in the analytic sample so that the results for the corrected variables would be comparable to those for whole blood lead” appeared incorrectly. “Geometric” should have been “arithmetic” to read “Each corrected variable was multiplied by the weighted arithmetic mean of hematocrit or hemoglobin, respectively, in the analytic sample so that the results for the corrected variables would be comparable to those for whole blood lead.”

## References

[1] Aoki Y Brody DJ Flegal KM Blood Lead and Other Metal Biomarkers as Risk Factors for Cardiovascular Disease Mortality. *Medicine*. 2016 95:e2223. 26735529 10.1097/MD.0000000000002223 PMC4706249

